# 支气管肺类癌的诊断及治疗

**DOI:** 10.3779/j.issn.1009-3419.2011.09.14

**Published:** 2011-09-20

**Authors:** 森 韦, 昕 李, 军 陈, 清华 周

**Affiliations:** 300052 天津，天津医科大学总医院，天津市肺癌研究所，天津市肺癌转移与肿瘤微环境实验室，天津医科大学总医院肺部肿瘤外科 Tianjin Key Laboratory of Lung Cancer Metastasis and Tumor Microenvironment, Tianjin Lung Cancer Institute, Department of Lung Cancer Surgery, Tianjin Medical University General Hospital, Tianjin 300052, China

**Keywords:** 类癌, 肺肿瘤, 典型类癌, 不典型类癌, Carcinoid, Lung neoplasms, Typical carcinoid, Atypical carcinoid

## Abstract

支气管肺类癌是罕见的肺部肿瘤，总体生长缓慢、预后尚可。根据其临床特征往往可以推测支气管肺类癌的诊断和亚型并指导治疗。其中年轻、CT表现为中心型肿瘤且无肺门或纵隔淋巴结肿大的病例，典型类癌的可能性较大。此亚型远处转移几率小，在手术治疗前除胸增强CT外可以不进行其它的常规术前分期检查。中心型肿瘤临床怀疑纵隔淋巴结累及或周围型肿瘤临床怀疑肺门纵隔淋巴结累及的病例，可能为不典型类癌。此亚型应做全面术前评估和分期。累及纵隔淋巴结的不典型类癌预后相对较差，应行多学科积极治疗。支气管肺类癌虽然其生物学特性不活跃，但均为恶性肿瘤，放化疗效果差，手术切除是最主要的治疗手段。彻底切除肿瘤、最大限度保留正常的肺组织是此类肺肿瘤外科治疗的基本目标。

## Abstract (en)

Bronchopulmonary carcinoid tumors are relatively uncommon neoplasms and typically benign and slow growing. However, more aggressive subtypes may develop early nodal and distant metastases. The clinical presentation allows prediction of the diagnosis and cell type and directs evaluation and treatment. Young age, central tumor, and no nodal enlargement are highly suggestive of typical carcinoid. These patients require no further diagnostic or staging tests beyond chest computed tomography and bronchoscopy before resection. For a high suspicion of atypical carcinoid (central cN2, peripheral cN1, 2), imaging for distant, metastases and mediastinoscopy is suggested, with multimodality treatment for an atypical carcinoid with N2 involvement. Because these tumors are generally resistant to chemotherapy, complete surgical resection is the primary form of therapy. Long-term survival for patients with typical carcinoid is excellent but is decreased in those with the atypical subtype. Complete tumor resection with preservation of uninvolved pulmonary parenchyma remains the fundamental goal in the surgical treatment of this unusual clinical entity.

支气管肺类癌是相对少见的恶性肿瘤，起源于支气管粘膜Kulcitsky细胞，年发病率只有百万分之2.3-2.8^[1]^。此类神经内分泌肿瘤总体预后尚可^[2, 3]^，其发生占所有类癌的1/4，构成原发性肺恶性肿瘤的2%-5%^[4]^。本综述回顾分析既往具有代表性的相关文献，总结支气管肺类癌的诊断及治疗，重点关注外科治疗方法和疗效。

## 1 支气管肺类癌概述

### 1.1 病理分型

肺神经内分泌肿瘤的组织学分类，依据对其生物学特性认识的程度不断地修正，最新的2004版世界卫生组织肺肿瘤分类法包括：典型类癌（typical carcinoid，TC； < 2个有丝分裂/高倍视野）、不典型类癌（atypical carcinoid，AC；2个-10个有丝分裂/高倍视野，坏死和结构破坏）、大细胞神经内分泌癌（large cell neuroendocrine carcinoma, LCNEC）和小细胞癌（small cell lung carcinoma, SCLC）^[5]^。原发性支气管肺类癌占可切除肺癌的0.4%-3%^[6]^，生物学特性虽不活跃，但仍具有侵袭和转移能力，常见胸外转移部位为肝、骨、脑和肾上腺。支气管肺类癌与LCNEC、SCLC均具有神经内分泌颗粒，但细胞生物学研究提示它们之间并无联系^[8, 9]^。

肿瘤TNM分期法是否同样适用于支气管肺类癌尚存争议，还未明确此类肿瘤TNM状态与患者长期生存之间的关系^[10]^。但是，一些新的数据^[11]^，包括IASLC（International Association for the Study of Lung Cancer）的回顾性分析，均表明对于肺神经内分泌肿瘤，TNM分期法同样有适用价值。

### 1.2 流行病学及临床表现

支气管肺类癌临床较为少见，在欧洲，一项大规模流行病学调查^[12]^显示年发病率约仅为十万分之0.2，而我国尚无明确发病率统计结果。支气管肺类癌患者年龄分布较广（4岁-86岁），TC人群大都50岁左右，男女比例均等^[9]^。AC更易发生于老年患者，但在 < 30岁的患者中，AC不足10%^[13]^。有文献^[14]^报道，多发神经内分泌肿瘤综合症Ⅰ型患者中约5%罹患支气管肺类癌。目前无证据表明吸烟与该病的发生直接相关。

大多数患者具有临床症状，在获得明确诊断之前症状往往可持续多年。所出现的症状与病变的大小和位置密切相关：较小的周围型病变通常无任何症状，只是在体检或胸部X线检查时偶尔发现；中央型病变引起的临床症状与其导致的支气管官腔狭窄程度和富血供的生物学特性有关，常见症状包括：咳嗽、咯血和同一肺段或肺叶的反复肺感染。长期支气管阻塞可以造成局部支气管扩张，其远端部分/全部肺组织毁损。此外，难治性哮喘、胸痛和胸膜腔积液也有报道。Modlin等^[15]^总结的病例中，40%的患者影像学检查结果表现为肺不张，25%为中心型团块，约30%为周围型团块，其余5%的患者胸部X线检查正常。

支气管肺类癌细胞可以产生一些氨基肽或类激素样的物质分泌入循环系统导致系列副肿瘤综合征。其中类癌综合症较为罕见，仅0.7%左右（17/2, 496）^[16, 17]^，和肿瘤组织释放血管活性物质尤其是5-羟色胺有关，临床多特征性表现为急性腹泻、肤色潮红、心悸、哮喘样症状和头痛。约80%的类癌肝转移患者有类癌综合症表现，且类癌综合症在AC中更为常见，与其易发生转移的生物学特性一致^[8]^。一项研究^[18]^指出126例支气管肺类癌中约25%的患者5-HIAA水平升高，一篇综述^[19]^同样指出5-HIAA水平升高约见于47%的类癌患者，但其它研究未见类似结论。所以这些不典型的症状和不确切的实验室检查结果的临床地位尚不明确，不能作为诊断支气管肺类癌的直接依据。

### 1.3 影像学特点

大多数支气管肺类癌患者胸部X线检查均有异常发现，但约10%漏诊。由于富血供特点，要区分中心型肿瘤、肺门或纵隔淋巴结、临近血管以及和不张的肺组织，胸部增强CT是基本的检查。周围型肿瘤往往表现为生长缓慢的均质、边界清楚的类圆形高密度影，约占支气管肺类癌的20%^[20]^。淋巴结增大的CT诊断假阳性率为20%-40%，假阴性率要低一些^[21]^。

核磁检查可作为术前评估的手段，以区分周围型病变和增强CT检查中显影的周围肺血管，并评估中心型病变侵犯纵隔血管与否。

因肿瘤组织对18-氟脱氧核糖的吸收代谢率较低，PET-CT诊断支气管肺类癌的灵敏度各家报道高低不一（14%-100%）^[22, 23]^，所以临床帮助不大。PET-CT一般不作为类癌纵隔侵犯和/或远隔部位转移评价的常规手段，因为其阳性及阴性结果的临床可靠性尚不明确。临床怀疑纵隔或者远处转移者应当通过活检证实。一种新的PET-CT检查介质，碳11标记的5-羟色胺血清素前体似乎能提高检查灵敏度（95%），能够分辨直径6 mm-30 mm的原发或转移性神经内分泌病变^[24]^。奥曲肽成像检查相对较为昂贵，故也不作为支气管肺类癌的常规检查。约1/3类癌生长抑素受体阴性，其奥曲肽影像检查结果不易和炎症区分，而且，几乎所有非小细胞肺癌（non-small cell lung cancer, NSCLC）、SCLC、肺炎、肉瘤样病变、肉芽肿或者淋巴瘤患者奥曲肽成像检查结果均为阳性^[25]^。一项研究^[25]^指出奥曲肽成像检查探测肝和骨转移的敏感性为65%-70%，但此结论仅可用于多发转移的病例。因此，与增强胸腹CT、骨扫描相比，奥曲肽成像检查不具备优势。

### 1.4 支气管镜检查特点

支气管镜检查使得术前获得准确组织病理诊断成为可能，因75%的支气管肺类癌起源于三级支气管开口。中央型类癌支气管镜检查通常表现为光滑的赤褐色或黄色新生物，其上被覆完整粘膜，而原发NSCLC或SCLC通常表现为不规则且易碎的新生物。所以气管镜下刷片或灌洗行细胞学检查价值不大，真正有意义的是深部活检。由于类癌肿瘤组织富血供特点，既往有镜下活检后出血需转行开胸止血的报道，但发生率非常低。总之，类癌的支气管镜检查是安全的^[26, 27]^，一组大宗病例分析^[28]^中，出血发生率为1.4%，且绝大部分能够自止，没有死亡病例。需要手术干预的大出血仅有4例，所占比例不足0.3%，而且此4例均发生于同一治疗中心，推测和该中心的治疗策略有关。

### 1.5 病理检查特点

因肺类癌固有生物学特性和严格的病理分型标准，即便取得足够活检标本，类癌的诊断准确率也仅有70%-80%，10%的类癌被误诊为SCLC或鳞状细胞癌^[29]^。切片的厚度和固定方法影响有丝分裂的计数，同样坏死也不易和浓缩凝固的细胞准确的区分，大多数研究认为得到精确的类癌诊断及分型非常困难。经胸肺穿刺诊断周围型类癌的准确性约为40%^[30]^，但是能够区分出的类癌亚型比例仅为10%-20%或更低^[30]^。术中冰冻切片检查诊断类癌的准确率为50%，其中25%被诊断为SCLC或NSCLC，余下的被诊断为各种良性或恶性肿瘤^[21]^。因此，当治疗策略被确定前，依临床特征作出的支气管肺类癌推断仍然重要，活检结果必须被慎重考虑。

## 2 诊断及评估

几乎所有的支气管肺类癌均为原发肿瘤，占所有类癌的25%。组织学上与类癌相近的、直径 < 5 mm的小结节被称作微小瘤，发生于7%-10%的类癌患者，尤其是妇女和老年患者，这些不应被当作转移，而且不能因此改变评估治疗方案^[31]^。对于大多数中心型支气管肺类癌，只有粘液表皮样癌与其有相似的临床特征，而粘液表皮样癌与支气管肺类癌的临床评估和治疗相近^[32]^。周围型支气管肺类癌的临床诊断很困难，其特点为圆形、边界清楚、生长缓慢以及低18-氟脱氧葡萄糖代谢水平。周围型类癌结节合并异位库兴氏综合征是具有特异性的^[33]^，虽然罕见，但此特点可以和其它周围型病变相区别。

怀疑支气管肺类癌的患者应当给予临床评估和增强CT检查，如无明确转移症状，PET-CT、颅脑核磁和骨扫描不必列为常规。全腹扫描和血检检测5-HIAA水平只在特殊情况下使用^[34]^。TC或AC的可能性可通过临床特征加以评估。较年轻类癌患者中约超过90%是TC，而在 > 50岁的类癌人群中，约20%-25%是AC。85%的中心型和50%-80%的周围型肿瘤是TC。诊断时约90%的TC和60%的AC均为pN0。15%的pN0和50%的pN1, 2为AC，性别差异无助于类癌亚型的诊断^[4]^。

### 2.1 中心型支气管肺类癌

年龄 < 30岁的患者，约95%的中心型cN0支气管肺类癌为TC。现有的文献资料不推荐对此类病例行进一步的影像学检查或有创的纵隔镜检查分期。诊断性活检也不可能改变治疗策略，因此，外科治疗是首选。中心型且有淋巴结肿大的支气管肺类癌中约50%病例被证实是AC。因AC具有较高的远处转移几率，进一步影像学检查可以尽可能发现隐蔽的转移性病灶。cN1和cN2的病例应当进行纵隔镜检查，或者进行电视辅助纵隔镜淋巴结活检，进而更准确地区分组织亚型。但也有文献认为cN1应首选纵隔镜检查，而cN2应首选远隔部位影像学扫描，排除远处转移。年龄 < 30岁的cN1患者，因病理亚型为TC可能性较大，可按cN0诊治。

### 2.2 周围型支气管肺类癌

约25%的cN0周围型支气管肺类癌是AC。细针穿刺活检或术中楔形切除冰冻病理检查，尤其是区分AC和TC时准确率不高。进一步的术前影像学检查或有创分期是不必要的，因为此类病例不超过10%病例会累及纵隔，只有不到5%的患者发生远处转移。手术治疗是首选。

大多数纵隔淋巴结肿大的周围型支气管肺类癌是AC。有创纵隔分期推荐用于cN1和cN2肿瘤。建议对于cN2病例，首先进行影像学远处转移评估，估计会有25%-30%的无症状远处转移病灶被发现。对于无症状的周围型cN1肿瘤，首先应行纵隔镜检查，若无纵隔淋巴结侵犯，外科治疗是首选，只有N2淋巴结累及时，才行远隔转移部位的影像学评估。

## 3 外科治疗策略及疗效

类癌的治疗首选外科手术切除^[35, 36]^，对于TC，外科治疗是真正有可能治愈的手段。外科治疗的目的是彻底切除肿瘤组织，并尽最大可能保留正常的肺组织，确保术后生活质量。对于中心型支气管肺类癌，选择支气管成形袖式切除是最佳选择。术中应行切缘冰冻病理检查以确定肿瘤切除彻底。虽然文献报道切缘阳性的病例仍可能获得长期生存，但术中病理证实的切缘阳性病例还是应当进行充分的手术切除，比如联合肺叶切除或全肺切除。TC术后可有较长的生存（5年和10年生存率均在90%及以上），只有3%-5%的病例术后复发^[37]^。AC的生存明显较差（5年及10年生存率分别为70%和50%），其中术后25%会复发，而且大多病例死于复发^[9]^。无论TC与AC，几乎所有的复发转移均累及远隔部位。淋巴结转移明显影响支气管肺类癌生存^[29]^。Mineo等^[38]^报道了一组数据，表明无论AC还是TC，均有较高的淋巴结微转移率，而区域淋巴结转移和术后局部复发密切相关。pN0与pN1, 2的TC患者的5年、10年生存率分别为90%和75%，而N1与N2之间差别很小。可切除TC的准确预后因素还未确定，一些学者认为TC直径与预后相关，但大多学者认为在T分期与预后的相关性方面，TC不如AC密切。事实上，对TC而言，不完全的切除是明确影响长期生存的预后因素。pN0的AC患者的5年、10年生存率为85%和70%，pN1, 2的AC患者的5年、10年生存率约为60%和50%^[39]^。Wurtz等^[40]^报道根治性切除加系统纵隔淋巴结清扫对支气管肺类癌，尤其是TC，发现了更多的非预期淋巴结转移，所以推荐无论TC还是AC，术中均应行淋巴结清扫而非采样。

外科切除支气管肺类癌，文献中各治疗中心对气管成形和局限性切除技术的应用各不相同。例如：袖式成形肺叶切除的比率在2%-14%之间，而全肺切除率在5%-27%之间^[28, 37, 41]^。

### 3.1 中心型cN0支气管肺类癌

大多中心型cN0类癌为TC。生存数据表明局限性切除治疗支气管肺类癌和扩大根治性切除的疗效是接近的，至少TC如此。支气管成形袖式切除术后获得良好的生活质量并长期生存已成共识。一项203例TC手术治疗结果的多因素分析研究^[37]^表明局限性切除的5年、10年、15年生存和肺叶及全肺切除疗效是接近的。几乎所有报道均证实局限性切除的中心型cN0支气管肺类癌复发率很低，但究竟切缘距肿瘤多远为安全距离尚未确定。有研究报道切缘距肿瘤≥5 mm时无局部复发，但是大多学者推荐术中冰冻病理检查确定切除范围。少数术后病理证实镜下切缘阳性的病例其无复发期为1年-10年^[42]^。

对于中心型cN0支气管肺类癌，系统纵隔淋巴结清扫地位尚不明确，推荐纵隔淋巴结采样来进行病理分期。cN0的TC不推荐进行纵隔淋巴结清扫，除非发现淋巴结被累及。对于cN0中心型AC，可行纵隔淋巴结清扫。一项研究^[35]^报道中心型cN0支气管肺类癌系统纵隔淋巴结清扫术后复发率较未清扫者低。

### 3.2 中心型cN1或cN2支气管肺类癌

中心型cN1（纵隔镜证实无N2, 3淋巴结累及）应当行手术切除以及纵隔淋巴结清扫。手术切除pN2的TC患者的术后生存很理想，对于pN2的AC患者的手术切除同样很重要，pN2支气管肺类癌纵隔淋巴结清扫是有益的。大宗病例分析结果^[9]^推荐pN1, 2的AC均应行纵隔淋巴结清扫。累及N2, 3病例（需纵隔镜确诊）的治疗策略应当依据其具体病理分型。纵隔镜检查取得的大块组织标本可提供可靠的病理分型结果。TC且有pN2的病例应行手术切除并行纵隔淋巴结清扫术。TC术后好的生存预后决定于其生物学特性，该类病例一般不需术后辅助化疗及放疗^[43]^。

N2淋巴结受累的AC的最佳治疗路径尚不明确。一般而言，手术加纵隔淋巴结清扫的局部治疗有益于生存，推测与其生物学特性有关。AC放疗的地位还未确定，而化疗的反应性与NSCLC相似。基于这些临床观察结果，一些治疗中心采用术前新辅助化疗的治疗策略。如果未进行术前治疗，pN1和pN2的AC患者均应给予辅助治疗，尽管还没有确切数据证明这样有益于生存^[44]^。

### 3.3 周围型cN0支气管肺类癌

大约75%的cN0周围型类癌是TC，但是此类病变理想的外科治疗方式是有争议的。Ferguson等^[45]^建议行肺段切除或楔形切除，因为局部复发几率低，远期生存好。因为针吸活检和术中冰冻病理都不能准确分清亚型，所以治疗时必须考虑到AC的可能性和因此带来的高淋巴结累及几率。因此建议周围型cN0的病例行肺叶切除。对于较为年轻的患者，楔形切除可以接受，因为其TC的可能性较大。少数几项研究报道了TC局部切除后的复发，其中一项研究^[46]^发现局部切除和肺叶切除患者的15年生存率相近，但肺叶切除者的20年生存率较局部切除好。

### 3.4 周围型cN1或cN2支气管肺类癌

多数周围型cN1支气管肺类癌是AC。中心型和周围型cN1的类癌治疗路径是一样的，首选手术切除加系统纵隔淋巴结清扫。对于AC，术后应给予辅助治疗；而对于TC，手术切除加纵隔淋巴结清扫的治疗效果令人满意。

大多数周围型cN2的病例为AC，纵隔镜检查可以确定其病理亚型。其治疗路径和cN2中心型类癌相近。AC根治性切除前推荐给予术前放疗和化疗。对于TC，切除加纵隔淋巴结清扫可以获得长期生存^[47]^。

## 4 化疗和放疗在治疗支气管肺类癌中的地位和作用

支气管肺类癌发病率低，不易行临床前瞻性研究。生物学特性决定根治性切除的TC即便区域淋巴结转移也不需要术后辅助治疗。文献^[44]^中支气管肺类癌术后辅助化疗方案和NSCLC类似，是以铂类为基础的联合化疗，但化疗反应性差。支气管肺类癌对放疗抵抗，一些学者^[48]^推荐对淋巴结转移的TC或AC行术后辅助放疗，但未能证明对长期生存有益。

## 5 随访

支气管肺类癌的生物特性决定其随访应至少20年，无论TC还是AC，术后前10年每年一次查体和胸片检查，后10年可以给予每两年一次检查，胸部CT和支气管镜检查不必列为常规，除非有中央性复发的可能性。TC大多数的复发发生于术后10年内，AC复发则多见于术后5年内，但是术后20年的复发也见报道^[49]^。

多数复发是远处复发转移，尚不清楚早期检查发现能否改变预后。偶尔局部复发病灶的治疗是可行有效的，但通常仅是姑息治疗。姑息治疗的生存一般是2年-3年，但是8年-10年也有报道^[9, 42]^。

## 6 总结

支气管肺类癌在全身类癌中占有一定比例，几乎所有的支气管肺类癌其生物学活性均较不活跃。对于中心型肿瘤，增强CT和气管镜检查是需要的，对于其它肺部肿瘤来说是常规的进一步影像学检查和激素水平检测则不是必须的。内镜检查活检是安全的，但其病理准确性还不尽如人意，尤其是TC。约90%的cN0类癌为TC，而cN1或cN2病例中有33%和66%可能为AC。AC中约25%表现为的周围型cN0肿瘤，50%的cN1和75%的cN2支气管肺类癌其准确病理为AC。几乎所有 < 30岁的病例为TC，而 > 50岁的病例中约25%是AC^[50]^。

可能为TC的病例不需进一步的影像学检查和有创检查，应当首选手术切除。R0的局限性切除远期疗效，比如支气管成形袖式切除对于TC是可以接受的。临床怀疑为淋巴结受累的TC，应给予纵隔镜检查和大块组织活检，仅当病理怀疑有N2淋巴结受累时，可给予进一步的影像学检查，排除远处转移。如果纵隔镜检查结果阴性，此类病例需行手术治疗加纵隔淋巴结清扫，加行辅助治疗^[9]^，但辅助治疗的临床地位尚未明确。高度怀疑的AC，影像学进一步探查远处转移病灶和纵隔镜检查是恰当的。N2淋巴结受累的AC需行多学科综合治疗。AC对化疗的反应性与NSCLC类似，而术后辅助放疗的意义仍有争论。因其特殊的生物活性，无论TC还是AC，外科治疗远期效果好，均推荐给予术后20年的随访^[51]^。
